# Revisiting the metallothionein genes polymorphisms and the risk of oral squamous cell carcinoma in a Brazilian population

**DOI:** 10.4317/medoral.24215

**Published:** 2020-12-19

**Authors:** Roberta Rezende Rosa, Marcelo Augusto Junior Garcia, Patrícia Terra Alves, Elisa Moraes Sousa, Letícia Santos Pimentel, Luciana de Paula Barbosa, Adriano Mota Loyola, Luiz Ricardo Goulart, Paula Cristina Batista de Faria, Sérgio Vitorino Cardoso

**Affiliations:** 1 Area of Pathology, School of Dentistry, Federal University of Uberlândia, MG, Brazil; 2 Laboratory of Nanobiotechnology, Genetic and Biochemical Institute, Federal University of Uberlândia, MG, Brazil

## Abstract

**Background:**

Metallothioneins (MTs) gene polymorphisms have been associated with the ability of free radical scavenging and detoxification of heavy metals leading to cancer development. Our aim was to revisit, in a Brazilian population, single-nucleotide polymorphisms (SNPs) of the MT gene family previously associated with oral squamous cell carcinoma (OSCC).

**Material and Methods:**

A case-control investigation with 28 OSCC patients and 45 controls was conducted, using conventional risk factors (tobacco use and alcohol consumption) as covariates. SNPs genotyping for rs8052334 (MT1B), rs964372 (MT1B), and rs1610216 (MT2A) was performed by PCR-RFLP, and SNPs for rs11076161 (MT1A) were analyzed by TaqMan assay.

**Results:**

The only SNP associated with increased risk for OSCC was the MT-1A AA genotype (OR = 4.7; *p* = 0.01). We have also evidenced for the first time a significant linkage disequilibrium between the SNPs of MT-2A and MT-1A in this population with the highest frequency (30%) of the unfavorable haplotype G/A/C/T (rs1610216 / rs11076161 / rs964372 / rs8052334) of MT gene polymorphisms (OR = 6.2; *p* = 0.04). Interestingly, after removing the effects of conventional risk factors, we have uncovered the significance of the AA genotype of the rs11076161 with increased odds of 19-fold higher towards OSCC development.

**Conclusions:**

This is the first demonstration that a significant linkage disequilibrium among gene polymorphisms of the MT family may affect susceptibility to oral cancer, which is conditioned by the G/A/C/T haplotype (rs1610216/rs11076161/rs964372/ rs8052334) and the MT-1A gene polymorphism has a potential clinical utility for the OSCC risk assessment.

** Key words:**Oral squamous cell carcinoma, polymorphism, metallothionein, oral cancer.

## Introduction

Oral squamous cell carcinoma (OSCC) is one of the most frequent cancers in the human population world-wide, with approximately two-thirds of all cases occurring in developing countries, which are associated with great morbidity and mortality (1). It has been widely accepted that the main risk factor for OSCC development is the long term exposure to carcinogens, such as tobacco and alcohol (2). There is a dose-response relationship between oral cancer risk and the amount of tobacco and alcohol consumed, with the combined impact being greater than their individual effects (3). The carcinogenesis of OSCC is a complex, multifocal process that occurs due to various genetic alterations in the squamous epithelium. Certain individuals may be more susceptible to acquiring cancer if their inherent ability to respond to oxidative and oncogenic stress is compromised (3). Despite the knowledge of carcinogens, there are individuals who are not exposed to these risk factors and develop cancer. Therefore, it is important to investigate additional susceptibility factors.

Metallothioneins (MTs) are cysteine-rich, metal-binding, low molecular weight, intracellular proteins that have multiple functions, such as free radical scavenging and detoxification of heavy metals (4). MTs are also known to participate in cell proliferation and apoptosis, which are important processes in carcinogenesis. Evidence suggests vital roles of MTs in tumor growth, differentiation, angiogenesis, metastasis, immune escape, and drug resistance (5). Human MT genes consist of four subfamilies, MT1 to MT4, found in chromosome 16 (4). In recent years, variations of MT expression have been linked to carcinogenesis, tumor progression, and resistance to cancer treatment (6). Studies have demonstrated increased expression of MT1/2 in various human tumors (7), including OSCC (8,9). Alterations in the expression of MT isoforms have been related to distinct behavior (metastasis and prognosis) for oral tumors (10).

In recent years, genome-wide association (GWA) studies have gained universal recognition as a powerful method to identify novel genetic markers related to increased susceptibility for diseases with complex underlying pathology, including malignancies (11).

Single nucleotide polymorphisms (SNPs) can alter gene expression or protein activity, and have been proposed to influence the susceptibility to cancer (11). Evidence suggests that genetic variations in MT may affect the risk to develop oral cancer (3,5). To our knowledge there is only one previous study (3) reporting association of SNPs in MT1 genes and the risk of OSCC, but none investigating SNPs in the MT2 gene.

The aim of this work was to investigate the major SNPs of the metallothionein gene family in a Brazilian population to revisit their association with oral squamous cell carcinoma (OSCC). Furthermore, we have also explored the haplotypic formation, which revealed a linkage disequilibrium among SNPs that will be discussed herein.

## Material and Methods

- Study population

This is a case-control study under the approval of the Human Research Ethics Committee of the Federal University of Uberlândia, Brazil. All participants signed written informed consent forms, in compliance with the principles of the Declaration of Helsinki.

All participants were recruited from the Dental Clinics of the Federal University of Uberlândia between January 2014 and December 2015. The group of cases comprised 28 non-blood related patients, with the following criteria for participation: I) histopathological diagnosis of intraoral squamous cell carcinoma (ICD-10-CM C01-06) recently provided at the institution, II) negative history of other types of primary cancers, and III) negative history of second primary or recurrent oral cancer. Patients with obvious cognitive impairment or those indicated for palliative care were excluded.

The control group consisted in 45 unrelated healthy volunteers, aged 40 years or more, who seek routine dental care at our institution. None of them had history of cancer at any site or oral soft tissue lesions.

All subjects were personally interviewed after initial diagnosis of OSCC to obtain information on age, gender, tobacco and alcohol consumption, and familial history of any type of cancer. Race/ethnicity was self-reported; smokers were defined as persons who had smoked more than 100 cigarettes in their lifetime; and drinkers were defined as persons who had used alcohol at least once a week for more than 1 year. Information regarding clinical staging and histopathological grading was obtained from patients’ medical files when available.

- SNPs Genotyping

Peripheral venous blood samples collected from volunteers were initially placed in tubes containing EDTA (ethylenediamine tetra-acetic acid) and stored at 4°C. Genomic DNA was isolated from whole blood using a Wizard® Genomic DNA Purification Kit (Promega, Madison, WI, USA), according to the manufacturer’s recommendations. DNA was resuspended in 100µL of DNA rehydration solution and stored at -20 ºC. The DNA concentration, purity and integrity were determined by spectrophotometry using NanoDropTM (Thermo Scientific) and by electrophoresis on 0.8% agarose gel with ethidium bromide stain.

This study considered four SNPs in MT genes: rs11076161 (first intron region of the MT1A), rs964372 (first intron region of MT-1B), rs8052334 (second intron region of MT1-B), and rs1610216 (5’ UTR region of MT2-A). Selection of polymorphisms reflected their potential effect on the gene transcription rate or previously reported association with the risk of cancer (3,12,13). All results were confirmed by duplicated assays.

For the SNP of MT1A gene, genotyping was performed by TaqMan® SNP genotyping assay (Applied Biosystems, USA, C_25996927_10), according to manufacturer’s instructions. The real-time PCR reaction consisted of an initial denaturation step at 95°C for 10min, followed by 40 cycles, each consisting 95°C for 15s, for denaturation step, and 60°C for 1min for annealing/extension.

The other SNPs were genotyped by PCR-restriction fragment length polymorphism (PCR-RFLP). The initial PCR reactions were performed using 2mM of MgCl2, 100µM of dNTP, 1.5U of Taq DNA Platinum Polymerase (Invitrogen, Burlington, Canada), 10X PCR buffer, 0.5µL of each primer (forward and reverse) and 100ng of genomic DNA in a final volume of 25µL. Primers, enzymes and products are described in Table 1 (14,15). The PCR conditions included an initial denaturation step at 95°C for 5 minutes, followed by 35 cycles, denaturation step at 95°C for 1 min, annealing step at 61-63°C for 1 min, extension step at 72°C for 1 min and final extension step at 72°C for 10 min. RFLP analysis was performed on 20µL: specific buffer, 2µg/µL bovine serum albumin (BSA) and each of the respective PCR products (1µg) by subjecting them to 5U of each restriction enzymes, during three hours at 25 or 37°C (for SmaI or HaeIII, respectively) according to the manufacturer’s protocol (both from Promega, Madison, USA)(14,15). DNA fragments were analyzed by electrophoresis on 2% agarose gel ethidium bromide-stained and visualized by a UV transluminator.

- Statistical data analysis

Allelic frequency distribution in case and control groups was analyzed by χ2 test. For this analysis, the reference allele was defined as the most frequent one (16). To verify if the allele distribution for each SNP was in Hardy-Weinberg equilibrium in healthy controls, we also used a χ2 test. To assess the association between each polymorphism and the risk of oral cancer, odds ratios (ORs) and their 95% conﬁdence intervals (CIs) were calculated using conditional logistic regression models adjusted for potential confounders (age, sex, smoking, drinking, and familial history of any type of cancer). These analyses were performed with BioEstat 5.0 software (Mamirauá, Belém, Brazil). Linkage disequilibrium (LD) coefficients were assessed for pairs of alleles between the two sites of MT polymorphisms, by using the default setting of Haploview software (17), it considered LOD > 2 as significant. Values of *p* below 0.05 were considered significant.

## Results

Clinical and demographic data of patients and controls are presented in Table 2. The mean of age for both groups was 54 years, and 80% of cases and controls were 45 years or older. Men, cigarette smokers, and alcohol drinkers were prevalent among cases, but there were not statically difference from controls, except for alcohol drinkers who presented 8.2-fold (OR = 8.2; *p* < 0.001) higher odds towards developing OSCC. Familial history of cancer was significantly more frequent in control subjects. This history was mainly related to breast, prostate or skin cancer, without reports of relatives with oral cancer in both case and control groups.

Allelic and genotypic distributions for MT polymorphisms are summarized in Table 3. For the SNP rs11076161 of the MT1A gene, carriers of the G allele were less prone to OSCC development than subjects with the AA genotype (*p* < 0.05). No significant association was observed between the SNPs rs1610216, rs964372 or rs8052334 and the OSCC occurrence.

**Figure 1:**
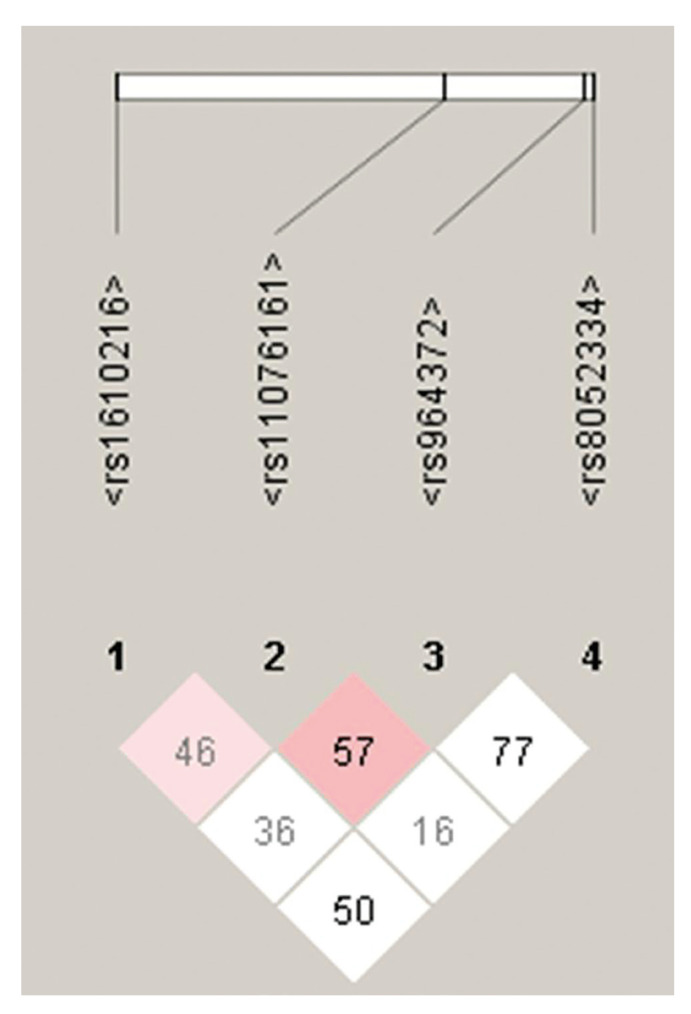
Linkage disequilibrium (LD) between MT-1polimorphisms. The numbers in the squares represent the pairwise D’ value as percentages. Areas in red (pink) indicate strong LD (LOD ≥ 2, D’ < 1); areas in light blue (or light gray) indicate moderate LD (LOD < 2, D’ = 1); white boxes indicate weak LD (LOD < 2, D’ < 1). This plot was generated by the Haploview program.

Pair-wise linkage disequilibrium (LD) analyses with these four SNPs of the MT genes by Haploview software (17), shown in Fig. 1, revealed LD among polymorphism, which was corroborated by the Hardy-Weinberg equilibrium analysis that demonstrated lack of fit among genotypic distributions, for the rs1610216. The haplotypic frequencies of rs1610216 / rs11076161 / rs964372 / rs8052334 in OSCC patients and controls are listed in Table 4. Only one haplotype (G / A / C / T) showed significant difference when compared with all others haplotypes, suggesting increased risk for OSCC.

**Table 1:**
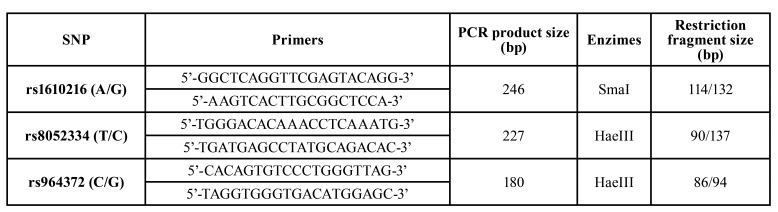
Primers, fragments, and restriction enzyme for each SNP (PCR-RFLP).

**Table 2:**
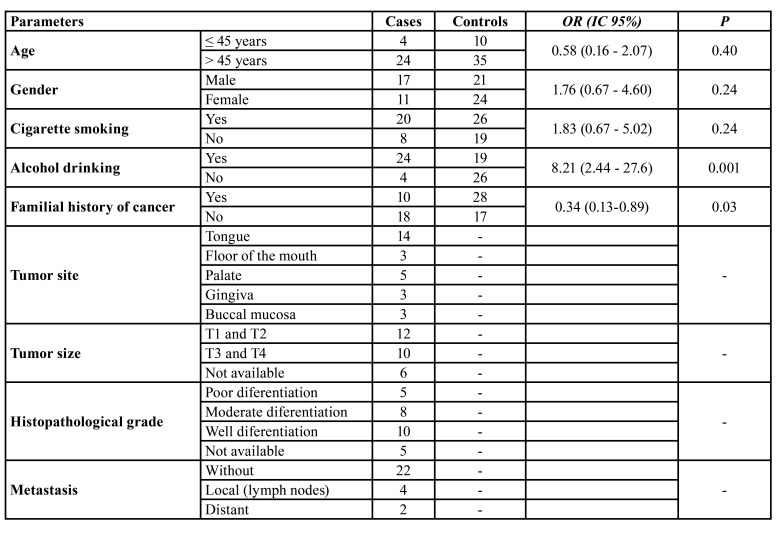
Clinical and demographic data of the patients.

**Table 3:**
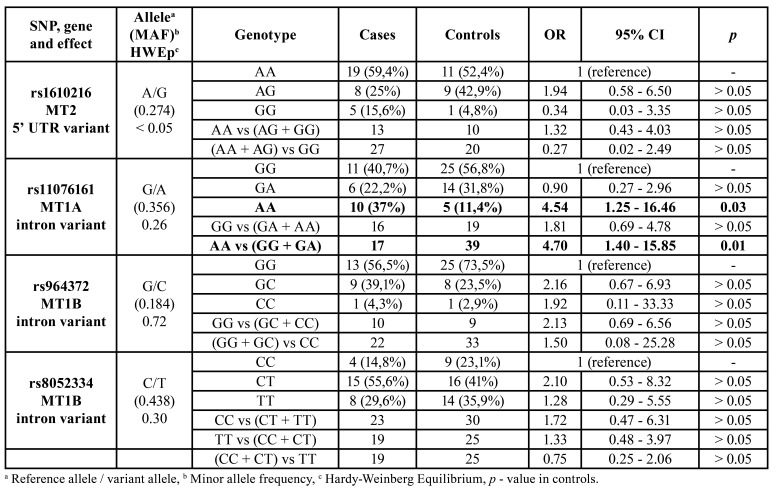
Summary estimates of the main effects of selected SNPs in MT gene region.

**Table 4:**
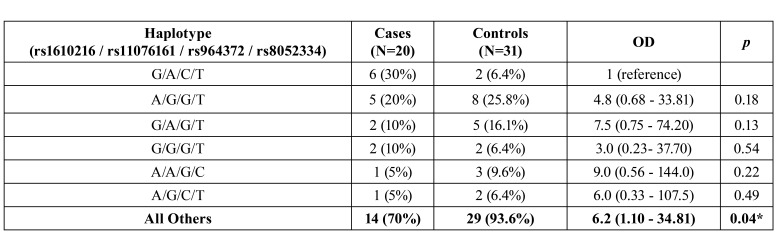
Frequencies of MT haplotypes in OSCC patients and matched control subjects.

## Discussion

Polymorphisms of MT genes have been associated with increased sensitivity to metal toxicity and oxygen-free radicals (18) through their reduced cellular activity, affecting cell proliferation, apoptosis, and response to oxidative stress, which may lead to chronic inflammation. Such continuous exposure to inflammatory cytokines may also induce carcinogenesis through different signaling pathways, and potentially influence susceptibility to various types of cancers (6,12,13,15,18-20). Furthermore, the SNPs can affect the MT mRNA transcription, and consequently the protein expression. Abnormal MT expression affect the p53 gene activation, since it is dependent of Zn ions modulated by MT, further leading to continued oxidative stress (21,22), which may further contribute to the carcinogenesis process (23). Familial history of cancer was prevalent among controls but not among cases. Oral cancer has not acknowledged as a heriTable disease. The reported tumors (breast, prostate, skin) have different etiology and have not strong association to the risk factors to oral cancer. Notwithstanding these facts, non-mutational genetic features must be investigated regarding its contribution to oral cancer.

Although the most important alterations in p53 are mutations in its gene - found in more than half of OSCC cases -, zinc removal by overexpressed MT may also be an important epigenetic event that links carcinogens to p53 disruption and subsequent malignant progression (3,21). We have previously demonstrated by immunohistochemistry a positive correlation between metallothionein and p53, in which a concomitant overexpression predicts shorter survival for patients with advanced OSCC (21). This is the second study that analyzes the association of SNPs in MT genes with the risk of OSCC development, but the first one that is not related with areca quid use. Areca nut consumption is also considered as a risk factor to OSCC, but it is more common in eastern countries. In a Taiwanese population (where 77.5% of patients reported areca quid chewing), a study revealed that both rs11076161 A, rs964372 C and rs7191779 C alleles were protective against OSCC, while rs8052394 A allele was associated with increased risk (3).

Differently from the previous study, we have shown that subjects with the AA genotype of the MT-1 rs11076161 are more predisposed to develop OSCC. Previous studies in a Chinese population corroborate our finding by revealing that the MT-1A rs11076161 was positively associated with type 2 diabetes with neuropathy (14) and with higher cadmium blood concentration related to renal dysfunction (24). It is important to mention that this specific SNP is a polymorphism in the first intron of MT1A. These data support the notion that introns may participate or mediate important biological activity. Introns may contain sequences that bind additional transcriptional enhancers or silencers, or codify regulatory RNAs that can modulate splicing signals, probably leading to non-functional proteins or to the production of different protein isoforms (25). However, the functional result of this polymorphism in the MT1-A gene has not been elucidated yet (26).

Briefly, the conflicting reports about the MT genes on cancer development led us to perform a reassessment on the polymorphisms of MT genes, and their impact on OSCC, which uncovered the significance of the AA genotype of the rs11076161 with increased odds of 19-fold higher towards OSCC development, after removing the confounding effects of conventional risk factors.

Also, the present study did not show a relationship among the SNPs rs1610216, rs964372, rs8052334 with OSCC, but have unveiled a linkage disequilibrium between SNPs, which may explain part of the contradictory reports about the SNPs and their relationship with the MT gene function. Our haplotype-based analyses showed that G/A/C/T haplotype (rs1610216 / rs11076161 / rs964372 / rs8052334) was significantly associated with 6-fold higher OSCC risk. This finding was consistent with the notion that haplotypes are more functionally relevant to certain diseases than single polymorphisms (27).

MT expression has been demonstrated to be induced by several factors including metals, glucocorticoids, lipopolysaccharides, steroid hormones, cytokines, inflammation, and stress (28). Moreover, some carcinogens, especially tobacco, also been considered as a triggers for MT overexpression (29).

Revisiting the literature, Wong *et al*. [2013] (20) evaluated the Taiwanese population with liver cancer and most of the population presented the AG genotype for the SNP rs11076161, data not observed in the Brazilian population, in which the GG genotype was the most prevalent. The variable gene polymorphisms among populations associated with the linkage disequilibrium and the accumulation of unfavorable haplotypes suggest that specific haplotypes and not individual metallothionein gene polymorphisms may be critical for predisposing an individual to OSCC.

In conclusion, our findings suggest that polymorphisms of the MT genes may affect susceptibility to oral cancer, but evidences indicated that the haplotype might be even more important for OSCC development due to MT dysfunction. Although confirmation of the present results in other populations are still necessary, MT genotypes and haplotypes may have a significant clinical utility in screening and for risk assessment of oral cancer, and haplotype-based analyses are required in different populations to uncover the functional effect of such polymorphisms.
